# Control of structural transition in FeSe_1−*x*_Te_*x*_ thin films by changing substrate materials

**DOI:** 10.1038/srep46653

**Published:** 2017-04-21

**Authors:** Yoshinori Imai, Yuichi Sawada, Fuyuki Nabeshima, Daisuke Asami, Masataka Kawai, Atsutaka Maeda

**Affiliations:** 1 Department of Basic Science, the University of Tokyo, Tokyo 153-8902, Japan; 2 Department of Physics, Tohoku University, Sendai 980-8578, Japan

## Abstract

Iron chalcogenide superconductors FeSe_1−*x*_Te*_x_* are important materials for investigating the relation be-tween the superconductivity and the orbital and/or electronic nematic order, because the end member material FeSe exhibits a structural transition without a magnetic phase transition. However, the phase separation occurs in the region of 0.1 ≤ *x* ≤ 0.4 for bulk samples, and it prevents the complete understanding of this system. Here, we report the successful fabrication of epitaxial thin films of FeSe_1−*x*_Te*_x_* with 0 ≤ *x* ≤ 0.7, which includes the phase-separation region, on LaAlO_3_ substrates via pulsed laser deposition. In the temperature dependences of differential resistivity for these films with 0 ≤ *x* ≤ 0.3, the dip- or peak- anomalies, which are well-known to be originated from the structural transition in FeSebulk samples, are observed at the characteristic temperatures, T*. The doping-temperature (*x*–*T*) phase diagram of FeSe_1−*x*_Te_*x*_ films clearly shows that T* decreases with increasing *x*, and that *T*_c_ suddenly changes at a certain Te content where T* disappears, which turns out to be commonly observed for both films on LaAlO_3_ and CaF_2_. These indicate the importance of controlling the structural transition to achieve high *T*_c_ in iron chalcogenides.

The discovery of iron-based superconductors has attracted much attention to fundamental studies and applications^1^. The iron-chalcogenide superconductors, FeSe^2^ and FeSe_1−*x*_Te_*x*_^3^ have the simplest crystal structure among the iron-based superconductors, composed of conducting planes alone. The superconducting transition temperature, *T*_*c*_, of FeSe is 8 K, and the partial substitution of Te for Se raises *T*_*c*_ up to ∼14 K. These values are rather low compared with other iron-based superconductors. However, the remarkable thing is the strong pressure dependence of *T*_*c*_; *T*_*c*_ of FeSe reaches ∼30 K under high pressure^4^,^5^. This fact suggests that FeSe thin films can have *T*_*c*_ values higher than those of bulk single crystals when in-plane strain is induced in the films. Indeed, FeSe films on CaF_2_ substrates, whose in-plane lattice parameters are much smaller than that of an FeSe bulk sample, show superconductivity with *T*_*c*_ of 15 K^6^,^7^,^8^, which are almost twice as large as that of an FeSe bulk sample. In addition, monolayer FeSe films on SrTiO_3_ substrates exhibit very high *T*_*c*_^9^. Although it is under debate whether both of these enhancements of *T*_*c*_ have the same origin or not, these results demonstrate that FeSe has a potential for a high *T*_*c*_ superconductor.

FeSe is also a unique material in iron-based superconductors in the sense that it exhibits a structural transition from tetragonal to orthorhombic at ∼90 K without a magnetic phase transition. The orbital-ordered and/or nematically-ordered state is observed below the structural transition temperature^10^,^11^. This is in contrast to other iron-based superconductors where a structural and a magnetic phase transition occur almost simultaneously. Therefore, iron-chalcogenide superconductors FeSe and Te-substituted Fese_1−*x*_Te_*x*_ are important materials for investigating the relation between the superconductivity and the orbital and/or electronic nematic order. However, it is known that single-phase FeS_1−*x*_Te_*x*_ bulk samples with 0.1 ≤ *x* ≤ 0.4 are not available because of a phase separation^3^, and this fact hindered the complete understanding of FeSe_1−*x*_Te_*x*_. Recently, we succeeded in obtaining FeSe_1−*x*_Te_*x*_ thin films on CaF_2_ substrates with these compositions by a pulsed laser deposition (PLD) method^12^. *T*_*c*_ of these films reaches 23 K at *x* = 0.2, and a sudden suppression of *T*_*c*_ is observed between *x* = 0.1–0.2. On the other hand, in the film preparation of FeSe_1−*x*_Te_*x*_ with whole range of *x* on MgO substrate^13^, only films with *x* = 0.1–0.4 have a very broad peak in the x-ray diffraction patterns, indicating the existence of the phase separation. Hence, it is of great interest whether the suppression of phase separation is realized only in the case of CaF_2_ substrate or not.

Here, we report the successful fabrication of epitaxial thin films of FeSe_1−*x*_Te*x* with *x* = 0–0.7 on LaAlO_3_ (LAO) substrate by the PLD method. The highest *T*_c_ of the films on LAO substrates reaches 19 K, which is also higher than that of bulk samples. The dip- or peak- behaviors, which are well-known to be originated from the structural transition in FeSe bulk samples^14^,^15^,^16^,^17^,^18^,^19^, are observed in the temperature dependences of differential resistivity for those films with 0 ≤ *x* ≤ 0.3. More importantly, in the doping-temperature (*x*–*T*) phase diagram of FeSe_1−*x*_Te_*x*_ thin films on LaAlO_3_, we observe a systematic decrease of its anomaly temperature in the resistivity data with increasing *x* and a sudden increase in *T*_c_ at *x* = 0.4 where the dip- or peak- anomaly disappears. These findings demonstrate that the sudden changes in *T*_c_ which are commonly observed for both films on LaAlO_3_ and CaF_2_ should be closely related to a structural transition regardless of the substrate materials.

## Results and Discussion

**Figure 1:**
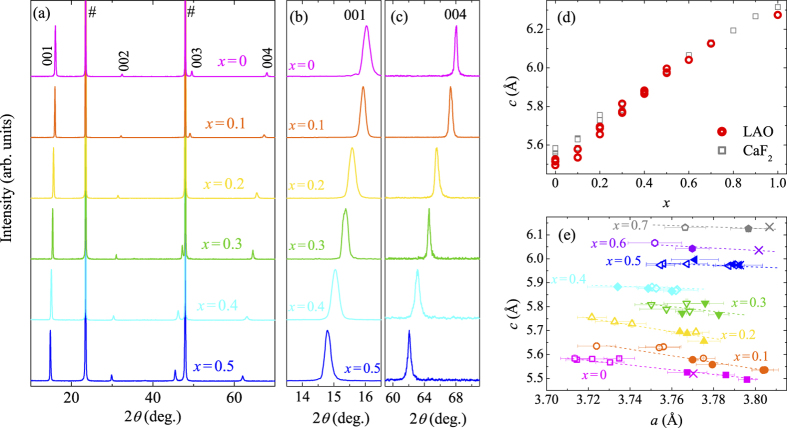
(**a**) XRD patterns for FeSe_1−*x*_Te_*x*_ thin films with *x* = 0–0.5 on LAO substrates. Sharps represent the peaks resulting from the substrates. Enlarged plots of (**a**) around the 001 and 004 peaks are shown in (**b**) and (**c**), respectively. (**d**) Dependence of the *c*-axis lengths for FeSe_1−*x*_Te_*x*_ thin films on the nominal Te content, *x*. Circles and squares represent the data for films on LAO and CaF_2_^8^,^12^,^20^, respectively. (**e**) Relationships between the *a*-axis and *c*-axis lengths in FeSe_1−*x*_Te_*x*_ films with *x* = 0–0.7. Closed and open symbols are the data of films on LAO and CaF_2_, respectively. The lattice parameters for FeSe_1−*x*_Te_*x*_ polycrystals with *x* = 0,0.5,0.6 and 0.7 are also plotted as the symbol of a cross. The dashed lines are guides for the eye. We cited the data of FeTe films on LAO and films on CaF_2_ from previous works^3^,^8^,^12^,^20^,^21^.

Figure 1a shows the X-ray diffraction (XRD) patterns of the FeSe_1−*x*_Te_*x*_ thin films with *x* = 0–0.5 fabricated on LAO substrates. Here and hereafter, the Te content *x* of the film represents the nominal Te composition of the polycrystalline target. These films show only the 00*l* reflections of the tetragonal PbO structure, indicating that the films are well-oriented along the *c*-axis. Figure 1b and c show the enlarged XRD patterns around the 001 and 004 peaks. The 2*θ* values of these peaks decrease with increasing *x* systematically, which is consistent with the dependence of *c* on *x* in FeSe_1−*x*_Te_*x*_. Here, it should be noted that there are neither splits nor broadening of the peaks even in the patterns of films with *x* = 0.1–0.4, contrary to the cases of bulk samples^3^ and films on MgO substrates^13^. These facts indicate that FeSe_1−*x*_Te_*x*_ films with *x* = 0–0.5 on LAO are single-phase samples, as films on CaF_2_ substrates^12^.

The *c*-axis lengths of FeSe_1−*x*_Te_*x*_ thin films on LAO are plotted as a function of *x* in Fig. 1d, where the data for films on CaF_2_^8^,^12^,^20^ are also plotted for comparison. The *c*-axis lengths of the films on LAO exhibit almost linear dependence on *x* including two end member materials, FeSe and FeTe^21^, as in the case of films on CaF_2_^12^. This suggests that Te contents of the grown films are nearly identical to the nominal values of the targets. Therefore, the above results show that the phase separation in FeSe_1−*x*_Te_*x*_ is successfully suppressed in films on LAO substrates, and that these suppressions are realized regardless of substrate materials in the film fabrication process. There are some variations of the *c*-axis lengths even in the films with the same composition, which is particularly notable at *x* = 0–0.2. We believe this fact is not due to the variation in the Te content^12^. Figure 1e shows the relation between the *a*-axis and the *c*-axis lengths in FeSe_1−*x*_Te_*x*_ films on LAO and CaF_2_^8^,^12^,^20^. For the samples with the same Te content, the *c*-axis and the *a*-axis lengths show a weak negative correlation regardless of the substrate materials, which cannot be explained by a variation in Te content because it should result in a positive correlation. This behavior observed in *c* v.s. *a* can be explained by considering the Poisson effect. Namely, the *c*-axis length slightly changes by the in-plane lattice strain depending on the film thickness or substrate materials. These results indicate that there is little variation in the Te content among the films with the same *x*.

**Figure 2:**
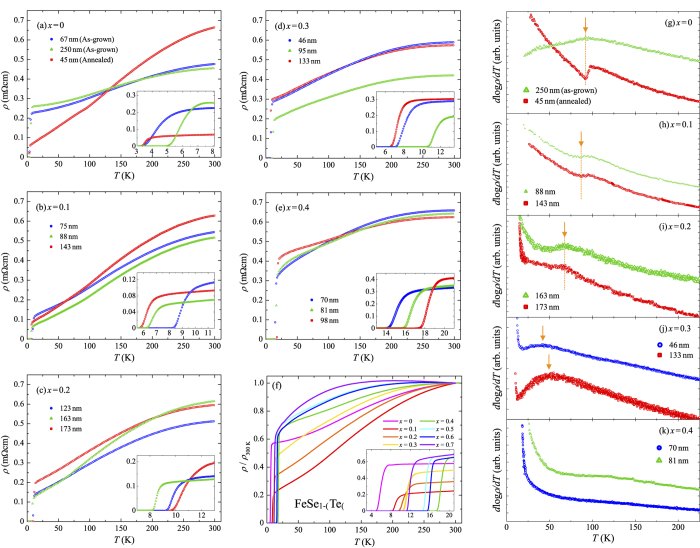
Temperature dependence of the electrical resistivity, *ρ*, of FeSe_1−*x*_Te_*x*_ thin films for (**a**) *x* = 0, (**b**) *x* = 0.1, (**c**) *x* = 0.2, (**d**) *x* = 0.3, and (**e**) *x* = 0.4 fabricated on LAO with different thicknesses. (**f**) Temperature dependence of the normalized resistivity for FeSe_1−*x*_Te_*x*_ films with the optimum film thickness for obtaining the highest *T*_c_ at each composition. The insets of (**a–f**) present enlarged views around *T*_c_. The temperature differential of a common logarithm of the electrical resistivity, *d*log*ρ/dT*, for FeSe_1−*x*_Te_*x*_ films with *x* = 0–0.4 is shown in (**g–k**).

Figure 2a–e show the temperature dependence of the electrical resistivity, *ρ*, of FeSe_1−*x*_Te_*x*_ thin films (*x* = 0–0.4) on LAO substrates with different film thicknesses. The residual resistivity ratio, *RRR*(= *ρ*(300 K)/*ρ*(20 K)), of these films decreases with increasing *x* except for *x* = 0. FeSe films on LAO with thickness of 67 and 250 nm show smaller *RRR* of approximately 2 than *x* = 0.1 (*RRR* ∼ 6) and an FeSe film on a CaF_2_ substrate (*RRR* ∼ 4)^8^. We found that an annealing process is effective for obtaining an FeSe film with a large *RRR* value. The red-open squares in Fig. 2a represent the temperature dependence of *ρ* for the FeSe film with thickness of 45 nm, which was kept at 450 °C for 15 minutes after film deposition in the same chamber. The *RRR* value for the annealed FeSe film is increased to 7. While an annealing improved the *RRR* values, *T*_c_ did not depend on them. Note that a kink anomaly is observed in the *ρ*–*T* curve of the annealed FeSe film below 100 K. The temperature dependence of the temperature differentials of log*ρ* for films with *x* = 0–0.4 are shown in Fig. 2g–k. The annealed FeSe film shows a clear dip structure in the curve of *d*log*ρ/dT* (Fig. 2g), and the characteristic temperature, T*, where *d*log*ρ/dT* takes its local minimum value (shown by a downward arrow in Fig. 2g) is estimated to be approximately 91 K for the annealed FeSe film. In FeSe bulk single crystals with very large *RRR* values, the kink in *ρ(T*) and the local minimum in the differential resistivity are known to be due to the structural transition from tetragonal to orthorhombic^14^,^15^,^16^,^17^. Thus, it is reasonable to regard that the structural transition occurs at T* in the annealed FeSe film. On the other hand, the as-grown FeSe films do not show a clear kink structure in *ρ(T*) (Fig. 2a); instead, a peak was observed in *d*log*ρ/dT* as shown in Fig. 2g. It is noteworthy that the temperature where the peak is observed in *d*log*ρ/dT* is almost the same as T* of the annealed FeSe film. Additionally, in FeSe poly crystals and single crystals with relatively small *RRR* values, it has been known that there is no clear kink in the *ρ*–*T* curves. Instead, a peak is observed in the differential resistivity^18^,^19^. This peak appears at approximately 90 K^18^,^19^, which is also found to be due to the structural transition. Therefore, not only the annealed film but also the as-grown films show the structural transition at T* as FeSe bulk samples. The differences of the behaviors in *ρ(T*) and *d*log*ρ/dT* around T* between the as-grown and annealed films are considered to result from the differences of *RRR* values. Thus, we define T* as the temperature where *d*log*ρ/dT* takes its local minimum (maximum) for films with large (small) *RRR* values, and try to estimate T* also at other compositions. Then, we obtained T* ~ 81 K for *x* = 0.1, T* ~ 64 K for *x* = 0.2, and T* ~ 44–48 K for *x* = 0.3 as shown by downward arrows in Fig. 2g–j. On the other hand, for FeSe_1−*x*_Te_*x*_ films with *x* = 0.4 (Fig. 2k), there is no peak- nor dip-structure in *d* log *ρ/dT*, which monotonically increases with decreasing temperature. This indicates that the structural transition does not occur at this composition.

**Figure 3:**
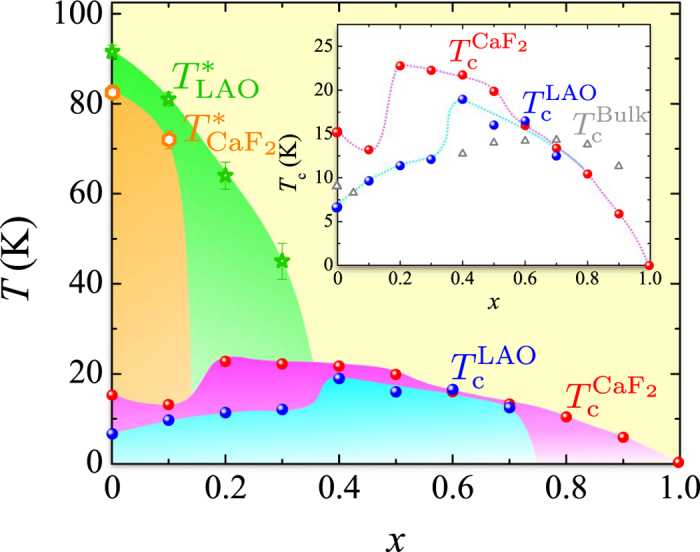
Phase diagram of FeSe_1−*x*_Te_*x*_ films. Blue circles (red circles) and light-green stars (orange hexagons) represent 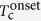 and T* of the FeSe_1−*x*_Te_*x*_ thin films fabricated on LAO (CaF_2_^8^,^12^,^20^), respectively. In the inset, the dependence of 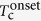 on Te content *x* is shown. The values of *T*_c_ for bulk samples estimated from the magnetic susceptibility measurements are shown as gray triangles^3^,^23^. The dashed curves in the inset are guides for the eye.

Next, we discuss *T*_c_ as a function of *x*. Since the superconducting transition temperature depends on the film thickness even for the same *x* as reported before^12^,^22^, we focus on the highest *T*_c_ for each *x*. Figure 2f shows the temperature dependence of the normalized resistivity for FeSe_1−*x*_Te_*x*_ films on LAO with the optimum film thickness for obtaining the highest *T*_c_ at each *x*. The onset temperature of the superconducting transition, 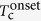, of the films in Fig. 2f and T* are plotted as a function of *x* in Fig. 3, where *T*_c_ and T* for films on CaF_2_^8^,^12^,^20^ (See the supporting information) and *T*_c_ of bulk samples^3^,^23^ are also plotted for comparison. Additionally, we set an error bar for considering slight deviations of T* at each compositions in Fig. 3. The FeSe_1−*x*_Te_*x*_ films on LAO show the highest *T*_c_ of 19 K at *x* = 0.4, which is higher than the maximum of *T*_c_ in FeSe_1−*x*_Te_*x*_ bulk samples at ambient pressure^3^,^23^, but lower than that in films on CaF_2_. We should note that Bellingeri *et al*. previously reported *T*_c_ of 21 K in FeSe_0.5_Te_0.5_ film on LAO^22^, which is higher than our *T*_c_ with the same composition, but is rather similar to *T*_c_ of our film with *x* = 0.4. The lattice parameters reported in ref. 22 are much smaller than those of FeSe_0.5_Te_0.5_ bulk samples and almost the same as those of our films with *x* = 0.4. Thus, we consider that the Te content of the film in ref. 22 is close to that of our film with *x* = 0.4, and that the optimum Te content for FeSe_1−*x*_Te_*x*_ films on LAO is *x* = 0.4. In Fig. 3, there are several common important features in *T*_c_ as a function of *x* between films on LAO and CaF_2_. At first, the highest *T*_c_ in FeSe_1−*x*_Te_*x*_ films is obtained at or near the “phase-separation region”. This enhancement of *T*_c_ seems to be consistent with a theory based on the electron-density wave fluctuation, which predicts that a high *T*_c_ phase is always next to the phase-separation region^24^. Second, the dependence of *T*_c_ on *x* suddenly changes at a certain Te content, *x*_c_, in films on both substrates; *T*_c_ increases with decreasing *x* for *x* ≥ *x*_c_, while *T*_c_ strongly suppressed for *x* < *x*_c_. The *x*_c_ values depend on the substrate materials; *x*_c_ = 0.4 for films on LAO and *x*_c_ = 0.2^12^ for films on CaF_2_. The increase of *T*_c_ with decreasing *x* for *x* ≥ *x*_c_ can be explained by an empirical law between *T*_c_ and the bond angle of chalcogen-iron-chalcogen, *α*^25^, as was discussed in the previous paper^12^. However, the sudden decrease of *T*_c_ is inconsistent with this empirical law. To understand the origin of this nontrivial behavior, we focus on the relation between *x*_c_ and T*. T* decreases with increasing *x*, and is observed only at *x* < *x*_c_. This result suggests that *x*_c_ corresponds to the Te content where T* becomes zero. The same thing is also valid for films on CaF_2_, while we observe T* only for *x* = 0 and 0.1. The highest *T*_c_ for films on each substrates is realized just at *x*_c_, that is, the Te content where T* disappears. The phase diagram of Fig. 3 indicates that the tellurium content necessary to suppress the structural transition varies depending on the kinds of substrate materials. Taking the similarity between films on CaF_2_ and LAO into consideration, the main origin of the sudden change in *T*_c_ at *x*_c_ is the presence or absence of the structural transition, and the control of the structural transition is essential to obtain higher *T*_c_ in iron chalcogenides. If we can suppress T* at smaller *x* in some way, for example by the introduction of some buffer layers, we expect higher *T*_c_. Looking at Fig. 3 carefully, the dependence of *T*_c_ on *x* at *x* < *x*_c_ differs between films on CaF_2_ and LAO. In terms of the empirical law of *α* v.s. *T*_c_, *T*_c_ should also increase with decreasing *x* at 0 ≤ *x* < *x*_c_, where the structural transition occurs at low temperatures. Indeed, *T*_c_ for films with *x* = 0 is higher than that with *x* = 0.1 in the case of CaF_2_ substrate. For films with *x* < *x*_c_ on LAO, however, *T*_c_ decreases with decreasing *x*, which is contrary to the empirical raw. This discrepancy might be due to the unexpected composition dependence of in-plane lattice parameters. In Fig. 1(d), the *c*-axis lengths for films on LaAlO_3_ with *x* = 0–0.3 systematically changes with *x*, which means that actual Te contents of the films vary with the nominal Te composition, *x*, as was already discussed. On the other hand, the *a*-axis lengths for films on LaAlO_3_ with *x* = 0–0.3 do not change almost at all as shown in Fig. 1(e). Since the Te substitution increases the lattice parameters both in the *a*- and *c*- directions for bulk samples, the small changes in the *a*-axis lengths of films on LAO with *x* = 0–0.3 show that the compressive strain is relatively weakened and that the local structures containing the bond angles for films on LaAlO_3_ with *x* = 0–0.3 are somewhat different from those for films with *x* > *x*_c_ on LaAlO_3_ and films on CaF_2_, which may be the origin of the characteristic dependence of *T*_c_ at *x* < *x*_c_. The reason for the small changes of the *a*-axis lengths for films on LaAlO_3_ with *x* = 0–0.3 is not clear at this moment. The measurement of the fine structure at the interface provides an important information on it, which is currently undergone. Finally, we briefly comment on the comparison between our phase diagram (Fig. 3) and the pressure-temperature (*P*–*T*) phase diagram^26^. The decrease of T* with increasing *x* in the phase diagram of Fig. 3 clearly shows that the structural transition is suppressed by the Te substitution of Se, which is consistent with the fact that the tetragonal phase is maintained at low temperatures in FeSe_0.5_Te_0.5_ bulk samples. On the other hand, the structural transition also disappears by introducing the physical pressure to FeSe. It is an open question why the structural transition is suppressed by both the physical pressure and the Te substitution, which introduces a negative chemical pressure to FeSe. Interestingly, however, the values of *T*_c_ suddenly increases at a pressure where a structural transition is suppressed according to the recently-reported *P*–*T* phase diagram of FeSe bulk single crystals^26^. We should note that the long-range magnetic order is induced after the suppression of the structural transition under high pressures in FeSe^26^, while there are no signatures of a magnetic transition in the transport properties of our films as shown in Fig. 2. Nevertheless, the rapid increase of *T*_c_ at the pressure, where a structural phase transition disappears, in FeSe is very similar to the jump of *T*_c_ at *x*_c_ seen in Fig. 3, which supports our conclusions.

In summary, we succeeded in fabricating epitaxial thin films of FeSe_1−*x*_Te_*x*_ with 0 ≤ *x* ≤ 0.7 by using LaAlO_3_ substrates, showing that thin film growth by PLD is effective for suppressing phase separation regardless of the substrate materials. In the temperature dependence of *ρ* and *d*log*ρ/dT*, there is an anomaly in films with *x* = 0–0.3 (LaAlO_3_) and *x* = 0–0.1 (CaF_2_), which corresponds to the structural transition. This means that the Te content necessary to suppress the structural transition can be controlled by changing the substrate materials. In our phase diagram, a sudden change of *T*_c_ is commonly observed at a certain Te content, which agrees with the Te content where a structural transition disappears in both films on LaAlO_3_ and CaF_2_. Therefore, this sudden change of *T*_c_ is related to the structural transition. Our result indicates that one of the key factors to realize a further increase of *T*_c_ in iron chalcogenides is the control of the structural transition.

## Methods

All of the films in this study were grown by the PLD method with a KrF laser^6^,^27^. FeSe_1−*x*_Te_*x*_ polycrystalline pellets (*x* = 0–0.7) were used as targets. The substrate temperature, and the base pressure were 300 °C, and 10^−7^ Torr, respectively. Commercially available single crystals of LAO (100) were used as the substrates, because LAO is one of the most suitable substrate materials among oxides for the film growth of FeSe_1−*x*_Te_*x*_^22^,^28^,^29^. These films were deposited in a six-terminal shape using a metal mask for transport measurements. The measured area was 1.2 mm long and 1.0 mm wide. The thicknesses of the grown films were measured using a Dektak 6 M stylus profiler. The crystal structures and the orientations of the films were characterized by X-ray diffraction (XRD) with Cu K*α* radiation at room temperature. The *a*-axis and the *c*-axis lengths were determined from the 204 and 00*l* reflections in XRD measurements, respectively. The electrical resistivity was measured by using the Physical Property Measurement System (PPMS, Quantum Design, Inc.) from 2 to 300 K.

## Additional Information

**How to cite this article:** Imai, Y. *et al*. Control of structural transition in FeSe_1−*x*_Te_*x*_ thin films by changing substrate materials. *Sci. Rep.* **7**, 46653; doi: 10.1038/srep46653 (2017).

**Publisher's note:** Springer Nature remains neutral with regard to jurisdictional claims in published maps and institutional affiliations.

## Supplementary Material

Supplementary Information
