# Supplementary material for: Control of structural transition in FeSe1−xTex thin films by changing substrate materials
Source: Sci Rep. 2017 Apr 21;7:46653. doi: 10.1038/srep46653 (PMC5399356; doi:10.1038/srep46653)
Supplement: Supplementary Information [file srep46653-s1.pdf]

Supplementary Information – Control of structural transition in $FeSe_{1-x}Te_x$ thin films by changing substrate materials

Yoshinori Imai[1,2,*], Yuichi Sawada[1], Fuyuki Nabeshima[1], Daisuke Asami[1], Masataka Kawai[1], and Atsutaka Maeda[1]

[1]Department of Basic Science, the University of Tokyo, Tokyo 153-8902, Japan
[2]Department of Physics, Tohoku University, Sendai 980-8578, Japan

[*]imai@tohoku.ac.jp

Electrical resistivity of $FeSe_{1-x}Te_x$ thin films on CaF_2 substrates

$FeSe_{1-x}Te_x$ thin films on CaF_2 substrates were fabricated by a pulsed laser deposition method[1]. Figure S1(a) shows the temperature dependence of the normalized resistivity, $\rho/\rho_{300\mathrm{K}}$, for $FeSe_{1-x}Te_x$ thin films with $x = 0 - 0.4$ on CaF_2 substrates[1–3]. The temperature differentials of common logarithms of the resistivity, $d\log\rho/dT$, for films with $x = 0 - 0.4$ are plotted as a function of temperature in Figs. S1(b)-S1(f). In films with $x = 0$ and $x = 0.1$, there is a clear kink in $d\log\rho/dT$ as described by the downward arrows in Fig. S1(b) and S1(c). This behavior is similar to the case of the as-grown FeSe film on $LaAlO_3$. Therefore, we estimate T^* as the temperature where a kink is observed in $d\log\rho/dT$; $T^* \sim 83$ K at $x = 0$, and $T^* \sim 73$ K at $x = 0.1$. In films with $x \geq 0.2$, on the other hand, $d\log\rho/dT$ increases monotonically with decreasing temperature, which indicates that a structural phase transition does not occur.

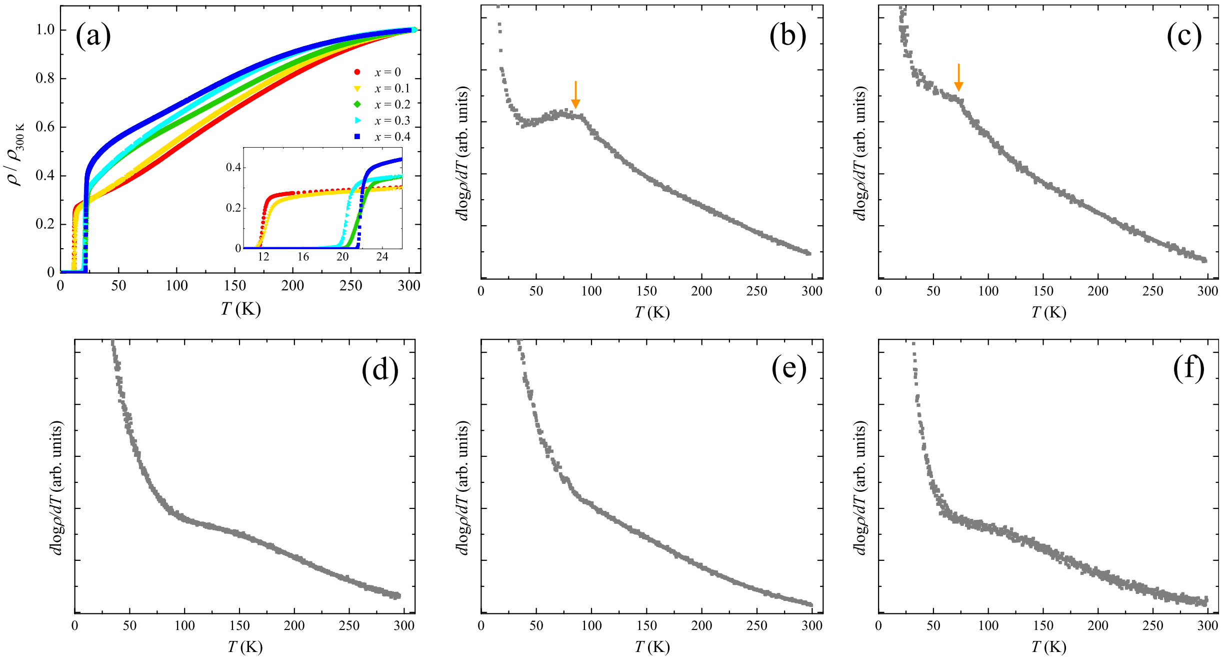

Supplementary Figure S1. (a) Temperature dependence of the normalized resistivity for $FeSe_{1-x}Te_x$ thin films with $x = 0 - 0.4$ on CaF_2 substrates.[1–3] The temperature differentials of common logarithms of resistivity are plotted as a function of temperature in (b) $x = 0$, (c) $x = 0.1$, (d) $x = 0.2$, (e) $x = 0.3$, and (f) $x = 0.4$.

[1] Y. Imai, Y. Sawada, F. Nabeshima, and A. Maeda, Proc. Natl. Acad. Sci. U.S.A. **112**, 1937 (2015).
[2] Y. Imai, Y. Sawada, D. Asami, F. Nabeshima, and A. Maeda, Physica C **530**, 24 (2016). 28th International Symposium on Superconductivity.
[3] A. Maeda, F. Nabeshima, H. Takahashi, T. Okada, Y. Imai, I. Tsukada, M. Hanawa, S. Komiya, and A. Ichinose, Appl. Sur. Sci. **312**, 43 (2014).
